# Integrating ToF-SIMS and machine learning reveals a mammary tumor-associated multi-ion signature

**DOI:** 10.1039/d6an01090c

**Published:** 2026-11-09

**Authors:** Auraya Manaprasertsak, Wei Tang, Caroline Lööf, Laila Azemovic, Sofie Mohlin, Catharina Hagerling, Kenneth J. Pienta, Julhash U. Kazi, Per Malmberg, Emma U. Hammarlund

**Affiliations:** a Department of Experimental Medical Science, Lund University Lund Sweden Auraya.manaprasertsak@med.lu.se; b Lund University Cancer Center, Lund University Lund Sweden; c Lund Stem Cell Center, Lund University Lund Sweden; d Department of Chemistry and Chemical Engineering, Chalmers University of Technology Gothenburg Sweden; e Division of Pediatrics, Department of Clinical Sciences, Lund University Lund Sweden; f The Cancer Ecology Center, Brady Urological Institute, Johns Hopkins School of Medicine Baltimore MD USA; g Multi-elemental Insights of Origins (MIO) Consortium Sweden

## Abstract

Cancer biomarkers should be specific, reproducible, and accessible from practical biological samples. Although omics-based approaches have substantially advanced biomarker discovery, many strategies still rely on individual molecules or limited molecular panels, which may not fully capture the complexity of tumor biology. Whether tumor-associated chemical signatures can improve cancer detection remains an open question. Here, we investigated whether a breast tumor-associated multi-ion signature could be detected in mammary gland tissue from the MMTV-PyMT breast cancer mouse model, as well as in liver, serum, and fur collected from the same animals. Low-mass ions (*m*/*z* 50–250) were analyzed using time-of-flight secondary ion mass spectrometry (ToF-SIMS) combined with multivariate analysis and machine learning. A robust mammary tumor multi-ion signature reliably distinguished tumor-bearing from healthy mice, with both partial least squares-discriminant analysis (PLS-DA) and random forest achieving an area under the receiver operating characteristic curve (AUC) of 0.997 under leave-one-animal-out cross-validation. Animal-label permutation testing confirmed that classification performance was significantly greater than expected by chance, and 98 of 313 ions differed significantly at the animal level. While liver and serum samples showed little evidence of tumor-associated discrimination, fur showed the highest classification performance and the greatest overlap in discriminative ions with mammary tissue. These findings demonstrate that low-mass ToF-SIMS enables robust identification of mammary tumor-associated multi-ion signatures, but that their detectability varies across biological matrices. Although the evidence in fur remained exploratory, it suggests that some tumor-associated chemical information may extend beyond the primary tumor and warrants further investigation of hair and related minimally invasive samples for cancer detection.

## Abstract 2

ToF-SIMS combined with machine learning reveals a robust mammary tumor-associated multi-ion signature. The signature strongly distinguishes mammary tumor tissue, while fur shows exploratory evidence of tumor-associated chemical information.

## Graphical abstract

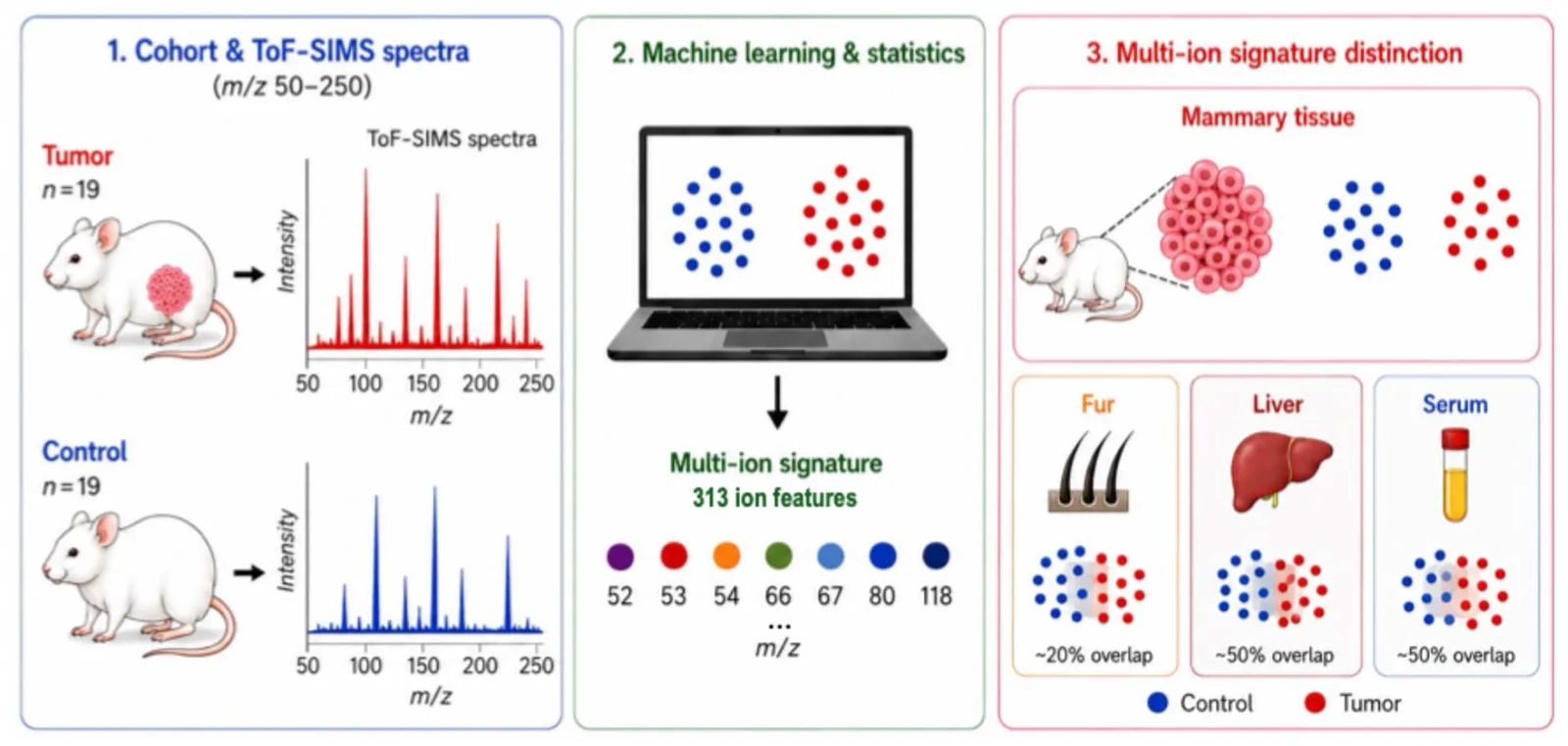

## Introduction

Cancer biomarkers are central to early detection, disease stratification, and disease monitoring, yet robust biomarkers for early cancer detection remain difficult to implement in practice.^1–3^ An ideal biomarker should be specific, reproducible, and accessible from clinically practical samples. Omics-based approaches using blood or urine have transformed biomarker discovery by enabling broad characterization of biological systems at genomic, proteomic, and metabolomic levels.^4^ However, many biomarker strategies still rely on individual molecules or limited panels that, in chemically dilute liquid biopsies, may miss components of early tumor biology.^5,6^ More fundamentally, it remains unclear how much tumor-associated and multi-analyte *chemical* information persists beyond the tumor itself and whether such information can be detected in accessible biological samples.

Multi-ion signatures offer an alternative approach for capturing the complex chemical states associated with disease. Time-of-flight secondary ion mass spectrometry (ToF-SIMS) provides rich chemical information from biological samples, including lipids, metabolites, and fragment ions, without prior molecular targeting or matrix application.^7–9^ Compared with matrix-assisted laser desorption/ionization (MALDI), ToF-SIMS requires minimal sample preparation and provides high spatial resolution, although it predominantly detects low-mass fragment ions.^10,11^ These characteristics make ToF-SIMS particularly well suited for probing tumor microenvironments, where coordinated biochemical alterations rather than isolated molecules may define disease states.^12–15^ However, ToF-SIMS datasets are inherently high-dimensional and sparse, requiring robust computational approaches to extract reproducible and biologically meaningful signatures.^12,16–18^

The detectability of tumor-associated multi-ion signatures outside breast tissue remains largely unexplored. Although multivariate analysis and machine learning have been widely applied to ToF-SIMS data,^15,19^ most studies have focused on within-tissue classification or localized chemical characterization.^20,21^ Comparatively little is known about whether tumor-associated multi-ion signatures remain detectable in other biological samples, or whether specific components of these signatures are preserved as chemical information moves beyond the tumor microenvironment. Understanding which elements of a tumor signature survive outside breast tissue is critical for developing minimally invasive biomarker strategies.

Here, we characterize a multi-ion signature of the murine mammary cancer model MMTV-PyMT and investigate whether this signature can be detected in serum, liver, and fur. Using ToF-SIMS combined with multivariate and machine learning analyses, we characterized low-mass ion (*m*/*z* 50–250) signatures in the MMTV-PyMT mouse model of breast cancer. We identified a robust tumor-associated multi-ion signature that reliably distinguished malignant from healthy mammary tissue. We then evaluated serum, liver, and fur independently to determine whether each biological sample contained their own tumor-associated discriminatory signal. This design distinguishes descriptive co-detection of ions across matrices from evidence of transferable classification, allowing the findings to be interpreted as tissue-specific signals.

## Methods

### Animal and tissue collection: mouse tissue

Female MMTV-PyMT transgenic mice and age-matched wild-type (WT) FVB/n controls were obtained from Scanbur (Denmark). All animal procedures were approved by the regional Ethics Committee for Animal Research (Dnr 5.8.18-15383/2019) and conducted in accordance with institutional and national guidelines. Mice were maintained until mammary tumors in the MMTV-PyMT cohort reached approximately 1.5 cm in diameter (10–15 weeks of age). At each tumor collection time point, an age-matched WT mouse was sacrificed as a control. Mammary glands were collected from 19 WT and 19 MMTV-PyMT mice, snap-frozen in liquid nitrogen, and stored at −80 °C until cryosectioning. In addition to mammary tissue, fur, liver, and serum samples were collected from the same animals. Liver and serum samples were stored at −80 °C until analysis, whereas fur samples collected from the dorsal region were stored at room temperature until analysis.

### Sample preparation prior to ToF-SIMS analysis

Mammary gland and liver tissues were cryosectioned at −25 °C using a cryostat microtome (Leica CM3050S, Germany) without embedding to minimize contamination. Fresh-frozen tissues were sectioned to a thickness of 10 μm, mounted onto glass slides, and stored at −80 °C until analysis. Prior to ToF-SIMS analysis, tissue sections were dehydrated using a freeze-dryer (Christ Alpha 1-2 LDplus, Germany). Fur samples were mounted onto glass slides using indium foil. Serum samples were prepared by droplet deposition onto clean glass slides and allowed to air dry prior to analysis. All glass slides were subsequently mounted on a top-mount sample holder.

### ToF-SIMS data acquisition

A total of 192 analysis spots were acquired from mammary gland samples obtained from 38 mice (19 MMTV-PyMT and 19 wild-type), corresponding to an average of approximately five analysis spots per mouse. In addition, 48 serum spectra (12 mice; 8 MMTV-PyMT, 4 wild-type), 40 liver spectra (10 mice; 6 MMTV-PyMT, 4 wild-type), and 61 fur spectra (20 mice; 10 MMTV-PyMT, 10 wild-type) were collected. For each animal, fur spectra were acquired from different locations along multiple individual hairs to account for local surface heterogeneity, with 3–4 analysis areas measured per animal. Spectra were acquired using a ToF-SIMS V instrument (ION-TOF GmbH, Münster, Germany) equipped with a 60 keV bismuth cluster ion source (Bi_3_^++^) operating in positive ion mode. The primary ion current was approximately 0.4 pA in spectrometry mode, corresponding to an ion dose of 4 × 10^12^ ions per cm^2^. Surface analysis was performed over an area of 500 × 500 μm^2^. Mass spectra were acquired over a mass range of *m*/*z* 0.5–800, yielding approximately 700 000 intensity measurements per spectrum at the instrument's native resolution. Initial internal calibration was performed using the characteristic positive-ion peaks [C]^+^, [CH]^+^, [CH_2_]^+^, [CH_3_]^+^, [C_5_H_15_PNO_4_]^+^, and [C_27_H_45_]^+^, which are commonly employed for positive-ion tissue analysis. Data acquisition and initial spectral processing were performed using SurfaceLab software (version 7.3; ION-TOF GmbH). Spectra were exported in ASCII format using the SurfaceLab export function with a binning factor of 1, a mass range of *m*/*z* 0–800, and dead-time correction applied.

### Spectral preprocessing

To reduce technical variability and enable quantitative comparison across samples, spectra were processed using mioXpektron,^22^ a modular Python-based workflow developed for preprocessing and analysis of ToF-SIMS spectral data. The workflow sequentially performs mass recalibration, denoising, baseline correction, normalization, peak detection and fitting, and peak alignment to generate a peak-area matrix for downstream statistical and machine-learning analyses. Mass recalibration used a multisegment model (breakpoints at *m*/*z* 50 and 150) with centroid-based peak detection; calibration anchors were selected by a bootstrap strategy with automatic fallback to *m*/*z*-based matching when fitting was unstable, and spectra were required to meet a maximum mass error of 100 ppm. Detector noise was suppressed using wavelet-based denoising. Each spectrum was first transformed using the Anscombe variance-stabilizing transform to stabilize the variance of the approximately Poisson-distributed ion counts. The transformed spectrum was decomposed over six levels using a discrete wavelet transform with the Daubechies-4 wavelet, and detail coefficients at each level were soft-thresholded using the BayesShrink threshold. To reduce the translation dependence of the discrete wavelet transform, denoising was performed over 16 circularly shifted copies of each spectrum and the reconstructed signals were averaged before transformation back to the original scale using an asymptotically unbiased inverse Anscombe transform. Wavelet-based approaches have previously been applied for denoising mass spectrometry and SIMS data.^23,24^ Chemical background was removed by asymmetric penalized-spline baseline correction (pspline_iarpls, lambda = 1 × 10^6^), and each spectrum was scaled to a constant total ion current (1 × 10^6^ counts) to correct for differences in total signal. Peaks were detected by continuous wavelet transform with Gaussian fitting and aligned across spectra by density-based clustering (DBSCAN). A peak that was not detected in a spectrum was recorded as zero, giving a peak-area matrix of 313 features (*m*/*z* 50–250). Analyses focused on the low-mass region (*m*/*z* 50–250), which showed higher intensity and reproducibility than higher masses (Fig. S1). Mass-calibration accuracy following preprocessing was evaluated across sample types (Fig. S2).

### Statistical and machine learning analysis

All inferential analyses treated the individual animal as the experimental unit; multiple analysis spots from the same animal were technical replicates.^25^ The dataset comprised 192 mammary-tissue spectra from 38 mice (19 MMTV-PyMT and 19 wild-type), 61 fur spectra from 20 mice (10 MMTV-PyMT, 10 wild-type), 48 serum spectra from 12 mice (8 MMTV-PyMT, 4 wild-type), and 40 liver spectra from 10 mice (6 MMTV-PyMT, 4 wild-type). The aligned peak-area table (313 features, *m*/*z* 50–250) defined the feature space; the 5% missing-value criterion refers to features not aligned across spectra and is distinct from the intensity-based detection below. All supervised preprocessing was performed strictly within cross-validation training partitions: in each training fold, features detected in fewer than 20% of training spectra were removed, non-detections (zero areas) were replaced by half the minimum positive training intensity, and intensities log_2_-transformed and Pareto-scaled using training parameters only, then applied unchanged to the held-out animal. Analyses focused on this low-mass region, which showed higher peak intensity and detection reproducibility than higher masses (Fig. S1). Principal component analysis of animal-mean profiles was used for visualization. Differential ions were identified by averaging spot intensities to one value per animal and comparing groups with a two-sided Mann–Whitney *U* test across animals, with Benjamini–Hochberg FDR control within each sample type;^26^ per-group detection frequencies are reported descriptively alongside intensity differences. Supervised classification used PLS-DA (five latent variables, fixed before resampling; sensitivity to two-to-eight components in SI) and random forests (500 trees; scikit-learn defaults). Models were evaluated by leave-one-animal-out cross-validation: each fold held out all spectra of one animal, fitted the full within-fold pipeline and classifier on the remaining animals only, and averaged the held-out spectra to one score per animal. As a pre-specified sensitivity analysis, classifiers were trained on individual spectra and on per-animal mean profiles. AUC was computed over animals with 95% confidence intervals from stratified bootstrap resampling of animals (2000 resamples; conditional on the fitted cross-validation). Significance used an animal-label permutation test re-running the entire pipeline^27^ (same 500-tree random forest) for a range of permutations 500–5000, statistic |AUC-0.5|; the eight tissue-by-model tests formed one Benjamini–Hochberg family. Because the tissue cohorts overlapped only partially, no formal between-tissue AUC test was performed; tissue-specific AUCs with confidence intervals are reported (Table 1). Discriminative ions were summarized by their cross-validation selection frequency: within each training fold, the top 15 ions by random-forest importance and the top 15 by the Mann–Whitney rank test were recorded, both for models trained on per-animal mean profiles (primary) and for models trained on individual spectra (Table S3), and ions selected in at least 90% of folds by both criteria were reported.

**Table 1:** Animal-level discrimination of MMTV-PyMT mice from wild-type mice by sample types. Leave-one-animal-out cross-validation (spot-trained, animal-scored); permutation *P* = 500 animal-label permutations, BH-adjusted across the eight tissue-by-model tests. Fur per-animal-mean sensitivity: PLS-DA AUC 0.88–0.93 (raw *P* 0.006–0.026; BH ∼ 0.10). Differential ions: animal-level Mann–Whitney, BH FDR < 0.05

Sample type	Mice (cancer/control)	Spectra	PLS-DA AUC [95% CI]	RF AUC [95% CI]	Permutation *P* (PLS/RF)	BH-adj. *P*
Mammary tissue	19/19	192	0.99 [0.98–1.00]	0.99 [0.98–1.00]	0.002/0.002	0.008
Fur	10/10	61	0.66 [0.39–0.91]	0.74 [0.48–1.00]	0.41/0.27	0.72–0.82
Liver	6/4	40	0.54 [0.13–1.00]	0.42 [0.04–0.79]	0.77/0.89	0.89
Serum	8/4	48	0.41 [0.09–0.75]	0.31 [0.00–0.66]	0.80/0.64	0.89

### Software and code availability

Analyses were performed in Python (version 3.13) using NumPy, pandas, SciPy, scikit-learn, statsmodels and Matplotlib. The preprocessing, cross-validation, permutation and figure code that regenerates reported statistic and figure from the peak-area table, are available at https://github.com/ershisan25/tofsims-breast. The analysis code and result tables are also archived on Zenodo (https://doi.org/10.5281/zenodo.21510507).^14^

## Results

### Standardized preprocessing enables reliable comparison of mammary tissue spectra

To enable reliable comparison across samples, ToF-SIMS spectra acquired from 192 mammary tissue analysis spots from 19 healthy and 19 tumor-bearing mice were subjected to a standardized preprocessing workflow to reduce technical variability and improve between-sample comparability. This process improved peak alignment across spectra, resulting in more consistent peak positions and reduced mass variation (Fig. 1A). Signal quality was further enhanced through noise reduction, baseline correction, and normalization, yielding cleaner spectra while preserving major spectral features (Fig. 1B and C). Peak areas were subsequently extracted for downstream analysis. Because the low-mass region (*m*/*z* 50–250) exhibited higher signal intensity and improved reproducibility than higher-mass regions, subsequent analyses focused on this spectral range to identify and characterize tumor-associated multi-ion signatures.

**Fig. 1:**
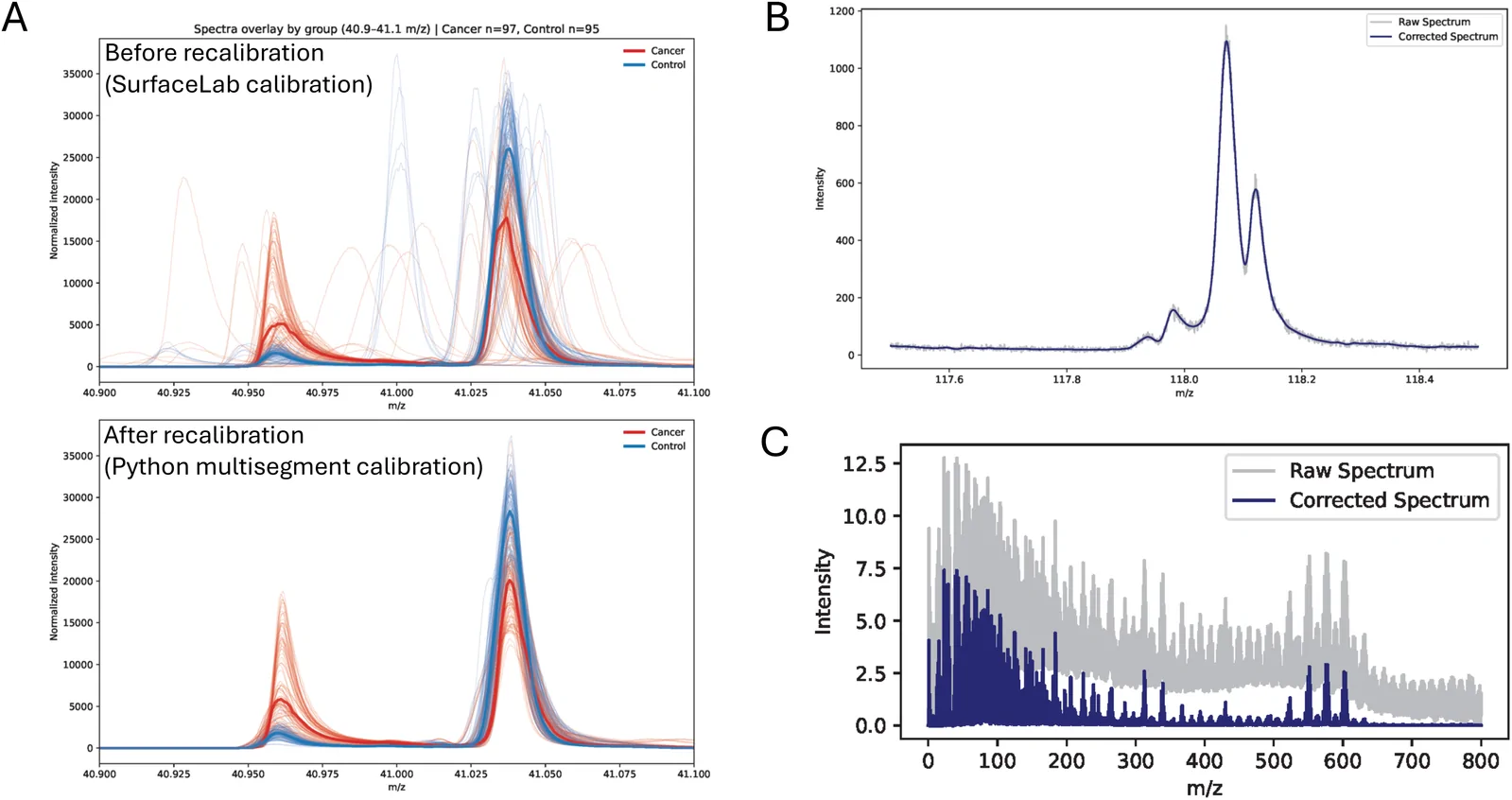
Preprocessing improves spectral quality and comparability. (A) Representative spectral region before and after mass recalibration using the multisegment calibration model. (B) Representative spectrum before and after wavelet-based denoising. (C) Representative spectrum before and after baseline correction and total ion current (TIC) normalization.

### A tumor-associated multi-ion signature distinguishes mammary tumor tissue from healthy mammary tissue

We next evaluated whether mammary tumors from MMTV-PyMT mice exhibited a distinct low-mass ToF-SIMS signature compared with wild-type controls. Animal-level analysis identified 98 significantly altered ions that differed significantly between groups (FDR < 0.05; Fig. 2A and B). Tumor tissue exhibited increased abundance of ions including *m*/*z* 118.07, 143.08, 145.09, and 174.87, whereas ions such as *m*/*z* 52.02, 53.02, 54.04, 65.03, 66.02, and 78.02 were reduced. The reduced ions were detected in all 19 tumor and 19 control animals but differed significantly in abundance between groups (Fig. 2B; Table S5). In contrast, *m*/*z* 118.07, 143.08, 145.09, and 174.87 were detected in 17 of 19 tumor animals but only 1–2 of 19 control animals (Table S5). PCA demonstrated partial separation between healthy and tumor tissues (Fig. 2C). PCA loadings further identified the ions contributing to this unsupervised separation (Fig. S7). PC1, which explained 42.2% of the variance, showed contributions from *m*/*z* 55.03, 67.05, 69.05, 81.02, 83.04, and 54.04 toward the control side and *m*/*z* 58.06, 86.10, 112.89, 114.90, 119.92, and 156.85 toward the tumor side. Supervised PLS-DA further improved group separation (Fig. 2D). Both PLS-DA and random forest achieved excellent animal-level classification performance under leave-one-animal-out cross-validation (AUC = 0.997; Fig. 2E and H). At the animal level, *m*/*z* 52.02, 53.02, 65.03, and 66.02 were selected in at least 90% of cross-validation folds by both the random forest and the rank-based criteria; the spot-level analysis (Table S3) additionally selected *m*/*z* 54.04, 67.05, 80.06, and 118.07. The complementary analyses consistently identified overlapping discriminatory features. PCA showed that tumor-associated differences were present in the overall spectral structure without using class labels, whereas PLS-DA and random forest identified the ions contributing most strongly to supervised discrimination. Several ions highlighted by the univariate and supervised analyses were repeatedly selected across cross-validation, supporting a coordinated multi-ion pattern rather than classification driven by a single ion.

**Fig. 2:**
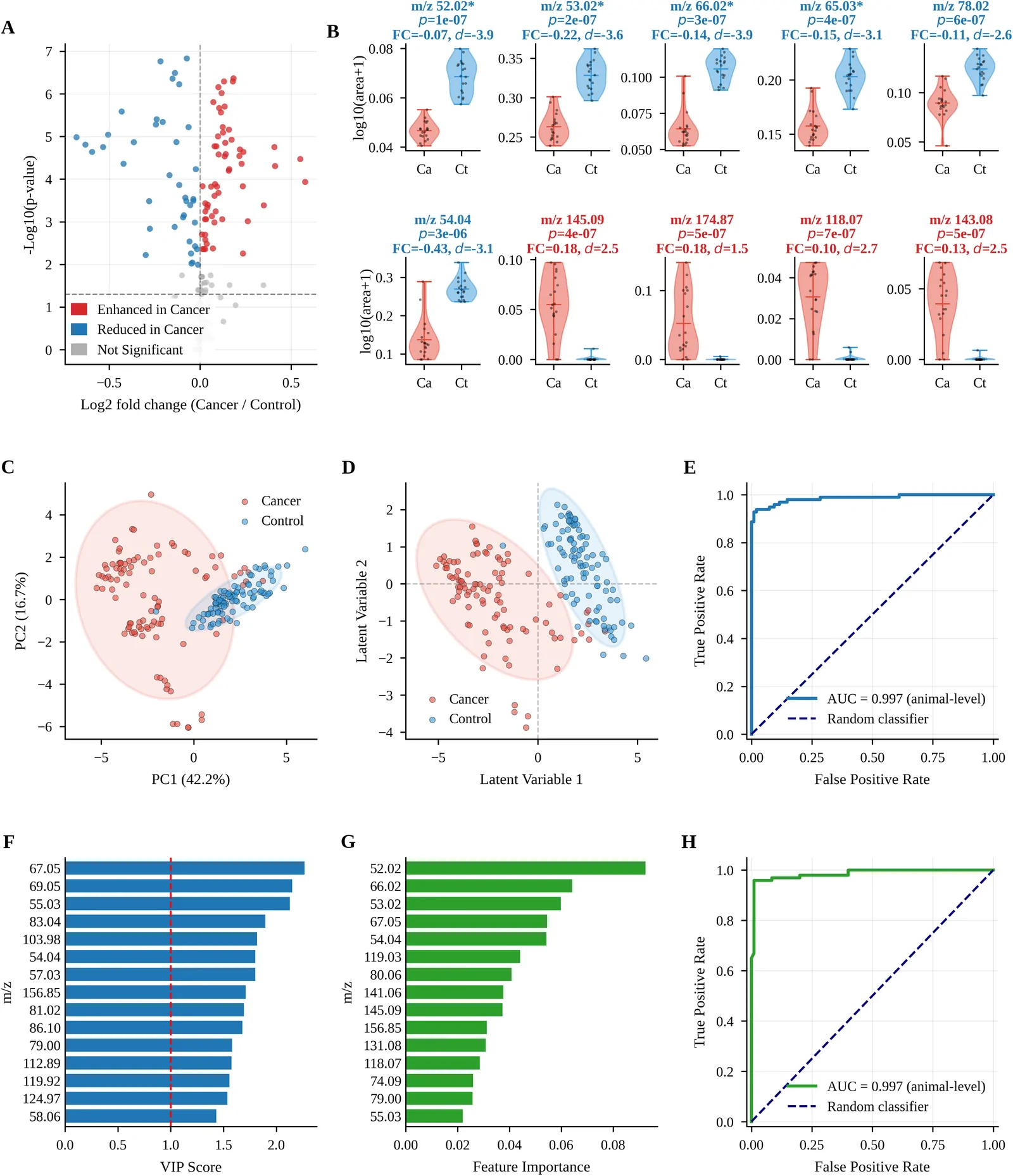
A stable tumor-associated multi-ion signature distinguishes mammary tumor tissue from healthy mammary tissue. (A) Volcano plot showing ions differentially abundant between healthy and tumor-bearing mammary tissue. (B) Violin plots of representative discriminative ions identified by univariate analysis. (C) Principal component analysis (PCA) of low-mass ToF-SIMS spectra showing the distribution of healthy and tumor samples. (D) Partial least squares-discriminant analysis (PLS-DA) score plot. (E) Receiver operating characteristic (ROC) curve for the PLS-DA model. (F) Variable importance in projection (VIP) scores of the top discriminative ions identified by PLS-DA. (G) Random forest feature-importance ranking of the top discriminative ions. (H) ROC curve for the Random forest classifier. Throughout, classification was performed at the level of the animal under leave-one-animal-out cross-validation (AUC 0.997 for both PLS-DA and random forest); 98 of 313 ions were differentially abundant at the animal level (two-sided Mann–Whitney, FDR < 0.05).

### Biological samples exhibit distinct chemical compositions but share common ions

To determine how tumor-associated signals may be distributed beyond mammary tissue, we first examined the intrinsic chemical relationships among mammary tissue, serum, liver, and fur samples. PCA of peak-area features revealed clustering primarily according to sample type (Fig. 3A and B). Along PC1 and PC2, fur formed a distinct cluster separated from the other sample types, whereas mammary tissue and liver occupied neighboring regions of the score space with partial overlap (Fig. 3A). Projection onto PC1 and PC3 further emphasized the separation of serum from mammary tissue, liver, and fur, reflecting its distinct chemical composition (Fig. 3B). PCA loading analysis identified the ions contributing most strongly to the separation among sample types (Fig. S6). The strongest positive and negative loadings, respectively, were *m*/*z* 115.05, 116.04, and 129.05 and *m*/*z* 92.93, 129.89, and 114.21 for PC1; *m*/*z* 134.78, 94.06, and 108.09 and *m*/*z* 66.02, 67.05, and 69.05 for PC2; and *m*/*z* 96.92, 140.90, and 138.90 and *m*/*z* 53.02, 55.03, and 54.04 for PC3. Examination of individual *m*/*z* features revealed that, although many ions were enriched in specific sample types, a subset was detected across mammary tissue, serum, liver, and fur (Fig. 3C). Together, these findings indicate that the biological samples possess distinct low-mass ion profiles while retaining a common set of ion features that provide a common chemical basis for comparing mammary tumor signatures across biological sample types.

**Fig. 3:**
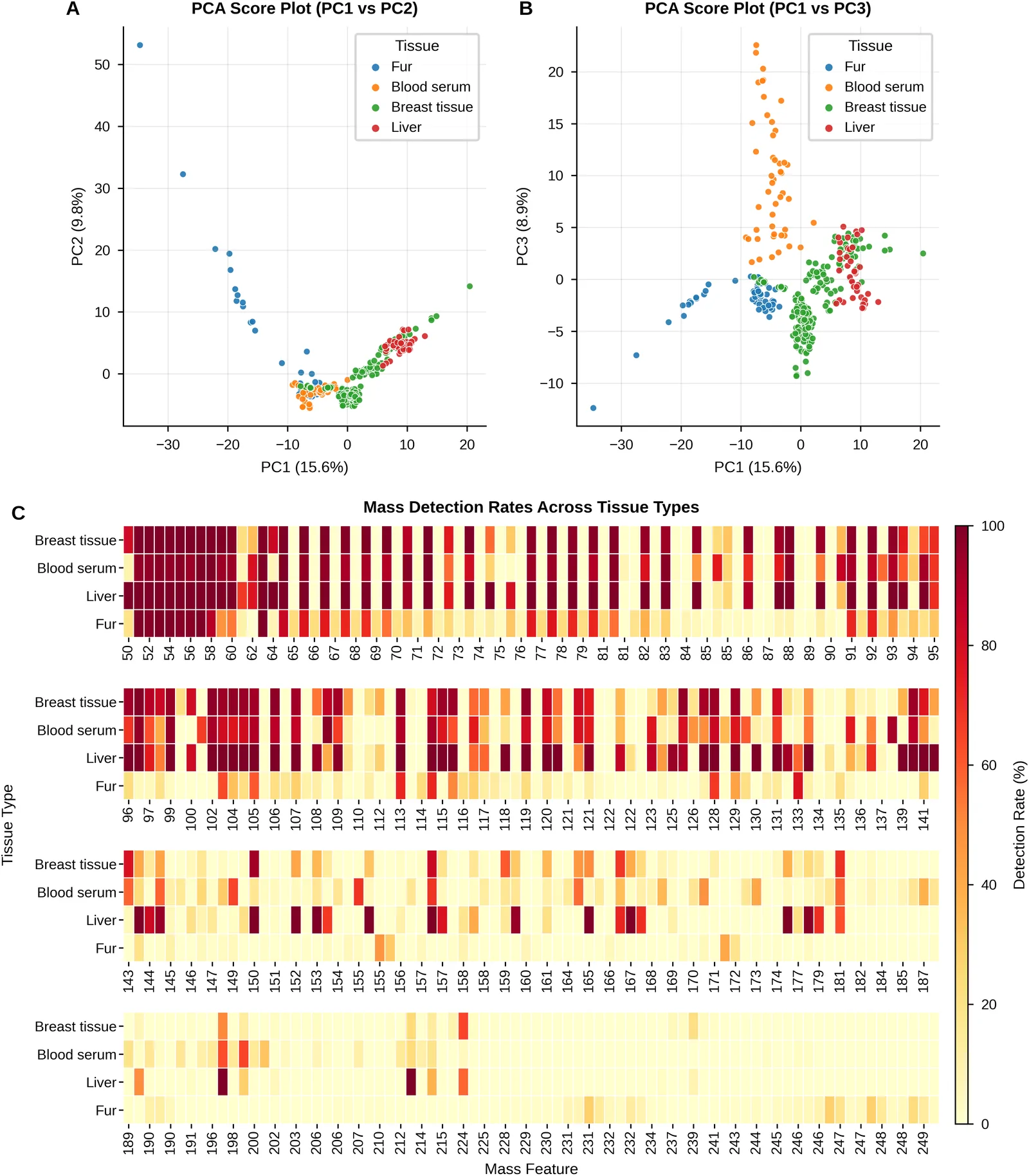
Distinct biological samples exhibit characteristic low-mass ToF-SIMS profiles. PCA score plots showing separation of mammary tissue, serum, liver, and fur samples along (A) PC1 and PC2 and (B) PC1 and PC3. (C) Heatmap of representative *m*/*z* features across mammary tissue, serum, liver, and fur samples.

### Beyond mammary tissue, fur provides the strongest evidence of tumor-associated signal

Having established a robust tumor-associated multi-ion signature in mammary tissue, we next investigated whether this discrimination extended to serum, liver, and fur collected from the same animals (Fig. 4; Table 1). Classification performance was markedly reduced in all three biological sample types compared with mammary tissue. Fur consistently showed the highest performance, whereas liver and serum remained close to random classification. Although exploratory analysis of per-animal mean fur spectra improved PLS-DA performance, this was not maintained after correction for multiple testing. No biological sample outside mammary tissue contained significant differential ions at the animal level. Together, these findings indicate that the mammary tumor signature was not reproduced outside breast tissue, although fur retained the strongest evidence of tumor-associated chemical information.

**Fig. 4:**
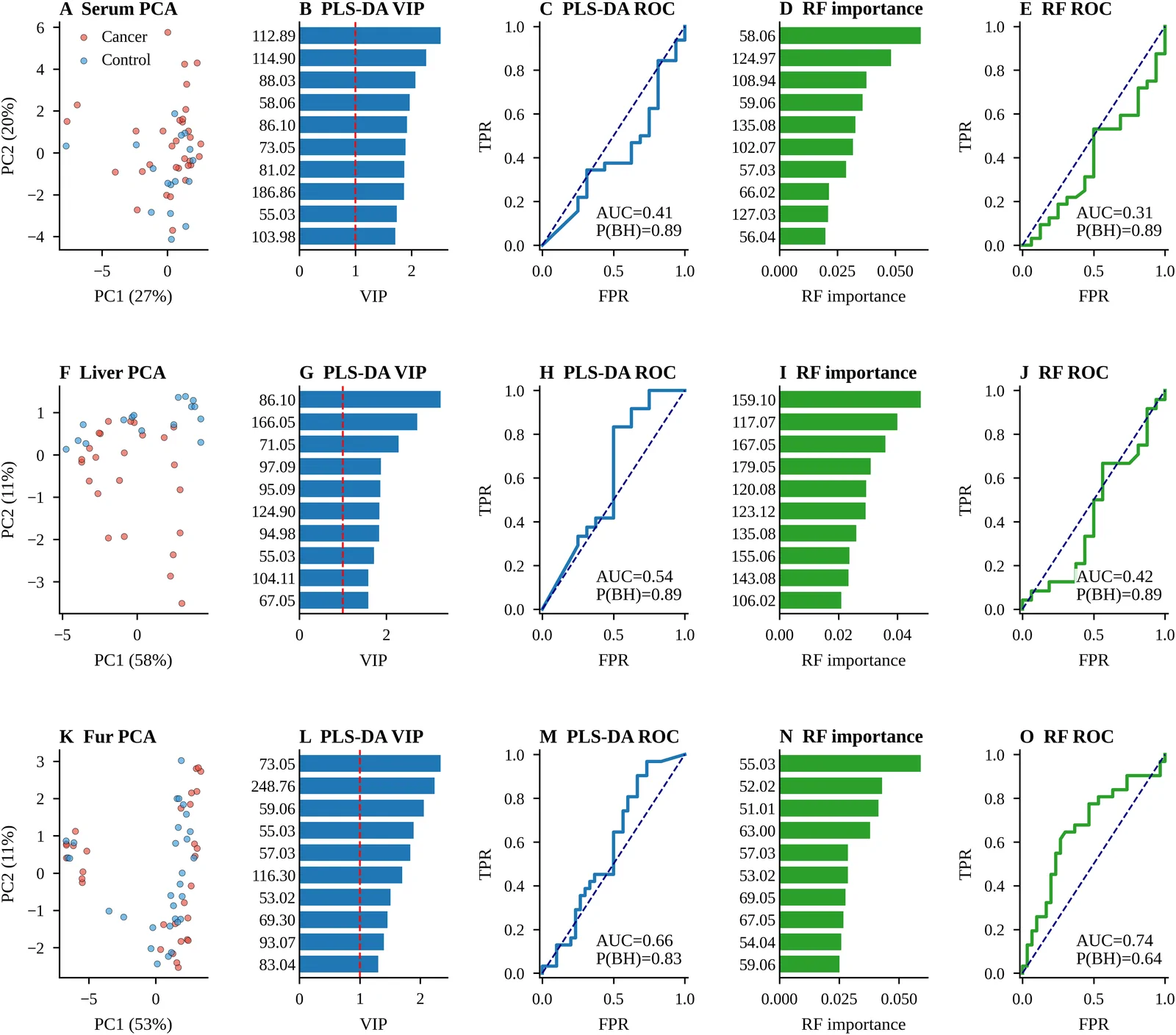
Discrimination of tumor-bearing from control animals in serum, liver, and fur. (A–E) PCA, PLS-DA, VIP scores, random forest feature importance, and animal-level random forest ROC curve for serum. (F–J) Corresponding analyses for liver. (K–O) Corresponding analyses for fur.

### Shared ions are co-detected across breast tissue, fur, liver, and serum

To determine which components of the mammary tumor-associated multi-ion signature were also detected in other biological samples, we compared the importance of shared ions across breast tissue, serum, liver, and fur using both VIP and random forest analyses (Fig. 5). Several discriminative ions identified in mammary tissue, including *m*/*z* 55, 57, 67, and 83, were detected across all four sample types, although their relative importance varied considerably. Additional overlap was observed between breast tissue and fur for ions including *m*/*z* 55, 57, 58, 81, 83, 86, 97, 104, and 113 in the VIP analysis, whereas random forest analysis identified overlap between breast tissue and serum involving *m*/*z* 52, 53, 54, 66, 67, and 69. These findings demonstrate that multiple components of the mammary tumor multi-ion signature are co-detected across biological sample types despite marked differences in their relative importance.

**Fig. 5:**
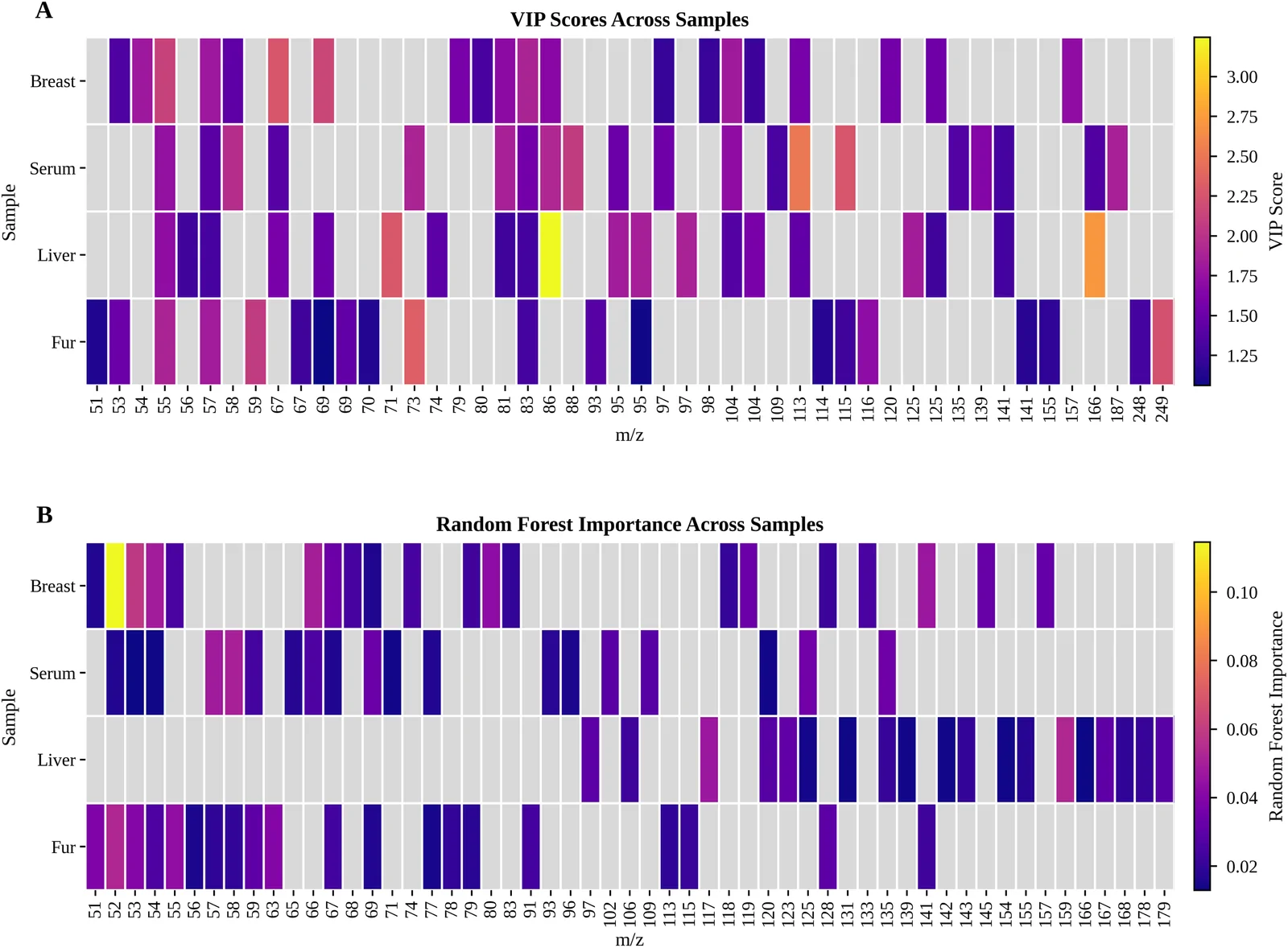
Shared ions across breast tissue, serum, liver, and fur samples. (A) Heatmap of variable importance in projection (VIP) scores for ions shared across sample types. (B) Heatmap of random forest feature importance scores for the same ions. Color intensity indicates the relative importance of each ion within the corresponding sample type; values are not directly comparable between sample types.

## Discussion

Mammary tumors in the MMTV-PyMT mouse model were characterized by a robust and reproducible low-mass multi-ion signature that distinguished tumor-bearing from healthy animals with excellent classification performance across independent analytical approaches. These findings demonstrate that coordinated low-mass ion patterns provide a stable representation of the tumor-associated chemical phenotype. Rather than relying on individual molecular biomarkers, multi-ion signatures integrate numerous chemical features that collectively reflect complex biological processes. Such an approach may provide greater robustness than single-analyte biomarkers, particularly in breast cancer where molecular and metabolic heterogeneity can limit the reproducibility of individual markers.^28,29^ Previous studies have demonstrated the value of supervised learning for extracting disease-associated information from complex SIMS datasets. Yun *et al.* applied PLS-DA to ToF-SIMS data to distinguish gastric cancer from normal tissue,^18^ while Park *et al.* used PLS-DA and multiblock PLS-DA to discriminate ovarian cancer from adjacent normal tissue.^30^ Random forest has more recently been applied to ToF-SIMS imaging data for classification and feature selection in complex biological samples.^31^ Together, these studies establish supervised learning as a useful framework for extracting discriminatory chemical information from high-dimensional SIMS data. The present study extends this approach by combining complementary PLS-DA and random forest models with animal-level cross-validation and feature-stability analysis to identify a reproducible mammary tumor-associated multi-ion signature.

Multi-ion signatures capture coordinated biochemical alterations rather than changes in individual molecular species. The reproducibility of the mammary tumor signature across independent statistical and machine learning approaches suggests that tumor-associated chemical information is distributed across numerous low-mass ions rather than being dominated by a single biomarker. Although definitive molecular identification is not possible from low-mass ToF-SIMS spectra alone, several discriminative ions have plausible candidate formulae and are consistent with biochemical processes known to be altered in breast cancer, including lipid remodeling, phospholipid metabolism, and broader metabolic reprogramming.^32–35^ For example, *m*/*z* 58 (C_3_H_8_N^+^), 86 (C_5_H_12_N^+^), and 104 (C_5_H_14_NO^+^) have been associated with phosphocholine-containing lipids, with *m*/*z* 104 commonly assigned to the choline head-group ion.^36–39^ These ions may originate from fragmentation of larger choline-containing biomolecules, including phosphatidylcholine and sphingomyelin, which are major components of cellular membranes. Changes in their relative intensities may therefore reflect alterations in membrane lipid composition or abundance, as well as differences in fragmentation behavior. Altered phosphocholine and membrane lipid metabolism are well-established features of breast cancer and are associated with the increased membrane synthesis and remodeling required for proliferating tumor cells.^32–35^ Several additional discriminative ions have plausible candidate formulae, including C_3_H_2_N^+^ (*m*/*z* 52), C_3_H_3_N^+^ (*m*/*z* 53), C_3_H_4_N^+^ (*m*/*z* 54), C_4_H_3_N^+^ (*m*/*z* 65), C_4_H_2_O^+^ or C_5_H_6_^+^ (*m*/*z* 66), and C_5_H_4_N^+^ (*m*/*z* 78).^40^ These low-mass ions may originate from fragmentation of various organic compounds, including proteins, amino acid-related molecules, and lipids. Alterations in amino acid metabolism have been widely reported in breast cancer,^41^ providing a possible biological context for their differential abundance. In addition, *m*/*z* 118 is consistent with C_8_H_8_N^+^, which may correspond to protonated indole ([M+H]^+^).^42^ This tentative assignment is of potential biological interest because indole-related chemistry is linked to tryptophan metabolism, a pathway frequently altered in cancer.^43^ Importantly, ToF-SIMS ion intensities are influenced by both molecular abundance and matrix-dependent ionization. Thus, the observed multi-ion pattern may reflect a combination of tumor-associated biochemical changes and differences in local tissue composition. These assignments remain tentative because low-mass ions may arise from fragmentation of multiple parent molecules, and their molecular origins cannot be established from the present ToF-SIMS spectra alone. Complementary structural approaches such as tandem mass spectrometry (MS/MS) would therefore be required for confident molecular identification.^44^ Taken together, the possible molecular origins of these ions provide a plausible chemical context for the observed signature, while the coordinated changes across the low-mass ion profile support its value as a reproducible discriminator of mammary tumor tissue within the studied model.

Fur provided the strongest evidence of a tumor-associated chemical signal among the biological samples examined. Although classification performance in fur was substantially lower than in mammary tissue and did not remain significant after correction for multiple testing, fur consistently outperformed both liver and serum across the analytical approaches applied. The heterogeneous nature of fur and the high surface sensitivity of ToF-SIMS should also be considered when interpreting these findings, as each measurement reflects localized surface chemistry rather than the bulk composition of fur. Although different locations along multiple individual fur hairs were analyzed from each animal, local variation in surface chemistry may contribute to the variability observed between measurements. Furthermore, several discriminative ions identified in mammary tissue were also detected in fur, suggesting that components of the mammary tumor-associated chemical profile remain detectable outside the primary tumor. These findings should be interpreted as exploratory rather than definitive, as no biological sample contained significant differential ions at the animal level. Nevertheless, the consistent performance of fur relative to liver and serum identifies it as the most promising biological samples investigated in this study for detecting tumor-associated chemical information beyond mammary tissue.

Hair may therefore represent an attractive complementary sample for investigating tumor-associated chemical signatures in future studies. Blood and serum are widely used in biomarker discovery because they are readily accessible, but they also represent highly dynamic biological environments in which tumor-derived molecules may be diluted, metabolized, or rapidly cleared. In contrast, several components of the mammary tumor-associated chemical profile were also detected on the fur surface, despite the considerably weaker overall signal. The mechanisms underlying this observation remain unknown. Although the fur analyzed here consisted of cut hair shafts and did not include the follicles, systemic metabolic changes during hair formation could potentially influence the chemical composition of the resulting hair. Chemical species may also be deposited onto the hair surface after growth. The relative contributions of these processes remain unknown, but the accessibility and ease of repeated collection of hair make it an interesting complementary sample for investigating tumor-associated chemical information. Interpretation of the fur findings should also consider biological differences between mice and humans. The relatively short duration of tumor development and faster growth cycle of murine fur may limit the accumulation of tumor-associated chemical information. Whether human hair retains more persistent tumor-associated chemical signatures remains an important question for future investigation. If confirmed, hair-based chemical profiling could complement existing omics-based biomarker approaches by enabling minimally invasive assessment of persistent tumor-associated chemical changes.

Low-mass ToF-SIMS enabled robust detection of tumor-associated multi-ion signatures but also limited direct molecular interpretation. Restricting the analysis to the low-mass region improved signal reproducibility and facilitated identification of fragment-based signatures with excellent classification performance. However, the fragmented nature of these spectra limits confident assignment of individual metabolites and lipid species. Complementary high-resolution techniques, such as OrbiSIMS and MALDI FT-ICR mass spectrometry, may improve molecular annotation while preserving the broader chemical context of the multi-ion signature.^11,45–47^ Integrating these complementary platforms could therefore strengthen the biological interpretation of future studies. In addition, unsupervised component-extraction approaches, such as non-negative matrix factorization (NMF) or multivariate curve resolution (MCR), could help resolve correlated ion patterns into chemically interpretable components and provide further insight into the underlying chemical origins of the multi-ion signature.

The biological scope of this study is limited by the use of a single mouse model and a restricted set of biological samples. The MMTV-PyMT model reproduces many molecular and pathological characteristics of aggressive breast cancer but does not capture the full biological heterogeneity observed across human breast cancers.^48–50^ As tumor development in this model is driven by the PyMT transgene, we also cannot exclude a contribution of transgene-associated effects to some of the observed chemical differences. Although the multi-ion signature was highly reproducible within this cohort, differences in tissue composition and model-specific characteristics may contribute to the observed chemical patterns, and independent validation is required to establish its broader applicability. Furthermore, the serum, liver, and fur cohorts were relatively small because these sample types were not collected systematically from the full mammary tissue cohort and were subsequently analyzed as a proof-of-concept extension. The corresponding analyses should therefore be regarded as exploratory and require validation in larger, independently powered cohorts. In addition, many discriminative ions remain chemically unresolved, limiting mechanistic interpretation despite the robustness of the overall multi-ion signature.

Human validation will ultimately determine the clinical relevance of these findings. Future studies should investigate whether similar multi-ion signatures can be detected in human breast cancer and whether hair exhibits comparable tumor-associated chemical information. Improved molecular annotation, integration with complementary mass spectrometry techniques, and longitudinal sampling will further clarify how these signatures emerge, evolve, and persist during disease progression. More broadly, understanding which components of a tumor-associated chemical signature remain detectable beyond the primary tumor may provide a foundation for developing more robust, context-aware, and minimally invasive biomarker strategies.

## Conclusion

Breast tumors in the MMTV-PyMT mouse model are characterized by a robust and reproducible multi-ion signature that can be resolved using low-mass ToF-SIMS spectra. While the strongest signal was observed in mammary tissue, fur provided the strongest evidence of tumor-associated chemical information among the biological samples examined, whereas liver and serum showed little evidence of discrimination. These findings indicate that tumor-associated chemical information is not equally detectable across biological matrices. Together, our results demonstrate the value of multi-ion signatures for capturing complex tumor-associated biochemical changes and highlight the importance of biological context when evaluating potential biomarker sources. Although the findings in fur remain exploratory, they identify hair as promising targets for future studies investigating minimally invasive chemical signatures of breast cancer.

## Author contributions

A. M.: conceptualization, data curation, formal analysis, investigation, methodology, visualization, writing – original draft, writing – review and editing; W. T.: formal analysis, methodology, visualization, writing – review and editing; C. L.: writing – review and editing. L. A.: writing – review and editing; S. M.: methodology, writing – review and editing; C. H.: methodology, writing – review and editing; K. J. P.: supervision, funding acquisition, methodology, writing – review and editing; J. U. K.: conceptualization, data curation, formal analysis, supervision, funding acquisition, investigation, methodology, visualization, writing – original draft, writing – review and editing; P. M.: conceptualization, resources, formal analysis, supervision, funding acquisition, investigation, methodology, writing – original draft, writing – review and editing; E. U. H.: conceptualization, resources, data curation, formal analysis, supervision, funding acquisition, validation, investigation, visualization, methodology, writing – original draft, project administration, writing – review and editing.

## Conflicts of interest

There are no conflicts to declare.

## Supplementary Material

AN-OLF-D6AN01090C-s001

AN-OLF-D6AN01090C-s002

AN-OLF-D6AN01090C-s003

AN-OLF-D6AN01090C-s004

AN-OLF-D6AN01090C-s005

AN-OLF-D6AN01090C-s006

AN-OLF-D6AN01090C-s007

AN-OLF-D6AN01090C-s008

## Data Availability

The raw ToF-SIMS spectra generated in this study are publicly available in Zenodo at https://doi.org/10.5281/zenodo.21506924. The analysis code and all preprocessed spectra used for statistical analyses and machine learning classification are available in a separate Zenodo repository at https://doi.org/10.5281/zenodo.21510507. Supplementary information (SI) is available. See DOI: https://doi.org/10.1039/d6an01090c.
