# Supplementary material for: Integrating ToF-SIMS and machine learning reveals a mammary tumor-associated multi-ion signature
Source: Analyst. 2026 Nov 9. Online ahead of print. doi: 10.1039/d6an01090c (PMC13746789; doi:10.1039/d6an01090c)

Supplementary Material (ESI) for Analyst.
This journal is © The Royal Society of Chemistry

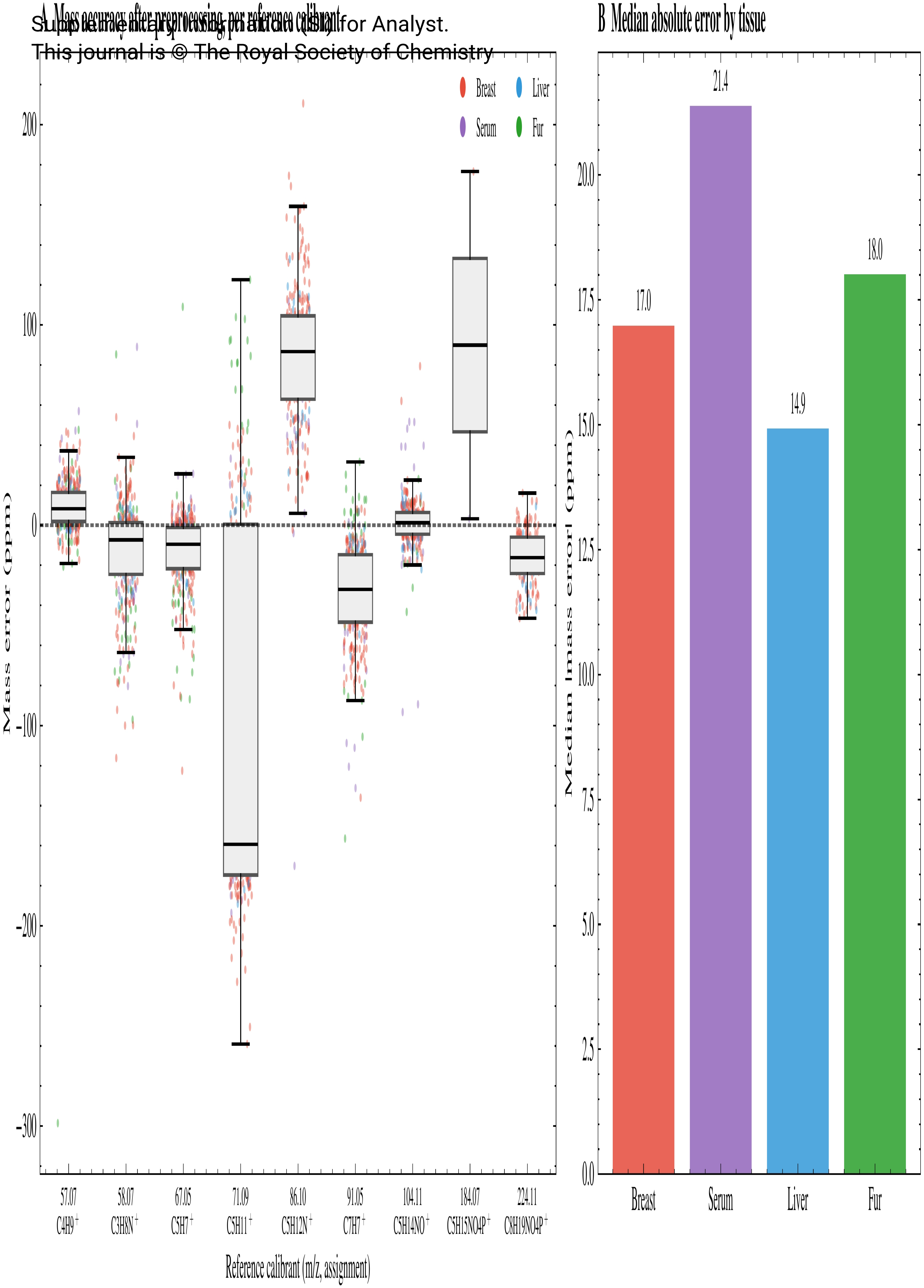

Supplement: Supplementary file 2 [file AN-OLF-D6AN01090C-s002.pdf]
