# Supplementary material for: Integrating ToF-SIMS and machine learning reveals a mammary tumor-associated multi-ion signature
Source: Analyst. 2026 Nov 9. Online ahead of print. doi: 10.1039/d6an01090c (PMC13746789; doi:10.1039/d6an01090c)
Supplement: Supplementary file 8 [file AN-OLF-D6AN01090C-s008.pdf]

Supplementary Information (SI) for Analyst.
This journal is © The Royal Society of Chemistry

# Supplementary Information

**Title**: Integrating ToF-SIMS and machine learning reveals a mammary tumor-associated multi-ion signature

**Authors:**

Auraya Manaprasertsak[a,b,c,g*], Wei Tang[a,b,c,g], Caroline Lööf[d,g], Laila Azemovic[a,b,c,g], Sofie Mohlin[b,c,e], Catharina Hagerling[a,b], Kenneth J. Pienta[f,g], Julhash U. Kazi[a,b,c,g], Per Malmberg[d,g], Emma U. Hammarlund[a,b,c,g]

**Affiliations**:

[a]Department of Experimental Medical Science, Lund University, Lund, Sweden

[b]Lund University Cancer Center, Lund University, Lund, Sweden

[c]Lund Stem Cell Center, Lund University, Lund, Sweden

[d]Department of Chemistry and Chemical Engineering, Chalmers University of Technology, Gothenburg, Sweden

[e]Division of Pediatrics, Department of Clinical Sciences, Lund University, Lund, Sweden

[f]The Cancer Ecology Center, Brady Urological Institute, Johns Hopkins School of Medicine, Baltimore, MD, USA

[g]Multi-elemental Insights of Origins (MIO) Consortium

*Correspondence: Auraya Manaprasertsak, Auraya.manaprasertsak@med.lu.se

## Supplementary Tables

**Table S1.** Animal and spectrum counts in the aligned m/z 50–250 feature table. Group counts are animals, not spectra.

| Sample type | Cancer animals | Control animals | Total animals | Spectra | Spectra per animal (min–max) |
|---|---|---|---|---|---|
| Mammary tissue | 19 | 19 | 38 | 192 | 5–6 |
| Fur | 10 | 10 | 20 | 61 | 3–4 |
| Liver | 6 | 4 | 10 | 40 | 4–4 |
| Serum | 8 | 4 | 12 | 48 | 4–4 |

**Table S2.** Primary mouse-level classification. AUC is calculated after leave-one-animal-out prediction and animal-level score aggregation. The 95% bootstrap CI resamples animal scores within class and is descriptive. Permutation p values are the confirmatory animal-label randomisation test; BH adjustment is over the eight primary tissue-by-model tests.

| Sample type | Model | Mice (C / Ctrl) | Spectra | AUC [95% CI] | Permutation p | BH p |
|---|---|---|---|---|---|---|
| Mammary tissue | PLS-DA | 19 / 19 | 192 | 0.997 [0.98, 1.00] | <0.001 | 0.002 |
| Mammary tissue | RandomForest | 19 / 19 | 192 | 0.997 [0.98, 1.00] | 0.001 | 0.004 |
| Fur | PLS-DA | 10 / 10 | 61 | 0.660 [0.39, 0.91] | 0.415 | 0.831 |
| Fur | RandomForest | 10 / 10 | 61 | 0.740 [0.48, 1.00] | 0.240 | 0.639 |
| Liver | PLS-DA | 6 / 4 | 40 | 0.542 [0.12, 1.00] | 0.800 | 0.890 |
| Liver | RandomForest | 6 / 4 | 40 | 0.417 [0.04, 0.79] | 0.890 | 0.890 |
| Serum | PLS-DA | 8 / 4 | 48 | 0.406 [0.09, 0.75] | 0.790 | 0.890 |
| Serum | RandomForest | 8 / 4 | 48 | 0.312 [0.00, 0.66] | 0.646 | 0.890 |

**Table S3.** Highest-frequency ions from cross-validation stability selection in mammary tissue (top 15 per analysis level). Within every LOAO training fold, the top 15 ions by random-forest importance and by univariate testing were recorded; an ion is called stable only if both frequencies are at least 0.90 (this defines the stable signature used in the main text). The complete 626-row selection-frequency table is supplied as Supplementary_Data_signature_stability.csv.

| Level | m/z | RF frequency | Univariate frequency | Mean frequency | Stable by both (>=0.90) |
|---|---|---|---|---|---|
| spot-level | 80.06 | 1.000 | 1.000 | 1.000 | Yes |
| spot-level | 52.02 | 1.000 | 1.000 | 1.000 | Yes |
| spot-level | 53.02 | 1.000 | 1.000 | 1.000 | Yes |
| spot-level | 54.04 | 1.000 | 1.000 | 1.000 | Yes |
| spot-level | 67.05 | 1.000 | 1.000 | 1.000 | Yes |
| spot-level | 66.02 | 1.000 | 1.000 | 1.000 | Yes |
| spot-level | 118.07 | 0.947 | 0.974 | 0.961 | Yes |
| spot-level | 55.03 | 0.632 | 0.947 | 0.789 | No |
| spot-level | 74.09 | 0.526 | 0.974 | 0.750 | No |
| spot-level | 78.02 | 0.184 | 1.000 | 0.592 | No |
| spot-level | 65.03 | 0.184 | 1.000 | 0.592 | No |
| spot-level | 69.05 | 0.447 | 0.684 | 0.566 | No |
| spot-level | 167.05 | 0.105 | 1.000 | 0.553 | No |
| spot-level | 141.06 | 0.974 | 0.053 | 0.513 | No |
| spot-level | 145.09 | 1.000 | 0.000 | 0.500 | No |
| animal-level | 52.02 | 1.000 | 1.000 | 1.000 | Yes |
| animal-level | 53.02 | 1.000 | 1.000 | 1.000 | Yes |
| animal-level | 66.02 | 1.000 | 1.000 | 1.000 | Yes |
| animal-level | 65.03 | 0.947 | 1.000 | 0.974 | Yes |
| animal-level | 145.09 | 0.895 | 1.000 | 0.948 | No |

| Level | m/z | RF frequency | Univariate frequency | Mean frequency | Stable by both (>=0.90) |
|---|---|---|---|---|---|
| animal-level | 118.07 | 0.737 | 1.000 | 0.869 | No |
| animal-level | 174.87 | 0.579 | 1.000 | 0.789 | No |
| animal-level | 54.04 | 0.342 | 0.947 | 0.644 | No |
| animal-level | 78.02 | 0.263 | 1.000 | 0.631 | No |
| animal-level | 152.03 | 0.184 | 0.974 | 0.579 | No |
| animal-level | 143.08 | 0.079 | 1.000 | 0.539 | No |
| animal-level | 139.04 | 0.026 | 1.000 | 0.513 | No |
| animal-level | 153.02 | 0.000 | 0.974 | 0.487 | No |
| animal-level | 133.10 | 0.026 | 0.947 | 0.486 | No |
| animal-level | 141.06 | 0.947 | 0.000 | 0.473 | No |

**Table S4.** Full exploratory sensitivity grid. min_det is the minimum within-training-fold detection fraction. Each raw p value is an animal-label permutation test (500 permutations for the peripheral tissues; the mammary p is floored at ≈0.002 by its near-perfect AUC). No grid cell is a separate confirmatory hypothesis test.

| Sample type | Training representation | Model | min_det | AUC | Raw p |
|---|---|---|---|---|---|
| Mammary tissue | animal-mean | PLS-DA | 0.0 | 0.99 | 0.002 |
| Mammary tissue | animal-mean | PLS-DA | 0.1 | 0.99 | 0.002 |
| Mammary tissue | animal-mean | PLS-DA | 0.2 | 0.99 | 0.002 |
| Mammary tissue | animal-mean | PLS-DA | 0.3 | 0.99 | 0.002 |
| Mammary tissue | animal-mean | RandomForest | 0.0 | 1.00 | 0.002 |
| Mammary tissue | animal-mean | RandomForest | 0.1 | 0.99 | 0.002 |
| Mammary tissue | animal-mean | RandomForest | 0.2 | 0.99 | 0.002 |
| Mammary tissue | animal-mean | RandomForest | 0.3 | 0.99 | 0.002 |
| Mammary tissue | spot | PLS-DA | 0.0 | 1.00 | 0.002 |
| Mammary tissue | spot | PLS-DA | 0.1 | 1.00 | 0.002 |
| Mammary tissue | spot | PLS-DA | 0.2 | 1.00 | 0.002 |
| Mammary tissue | spot | PLS-DA | 0.3 | 1.00 | 0.002 |
| Mammary tissue | spot | RandomForest | 0.0 | 1.00 | 0.002 |
| Mammary tissue | spot | RandomForest | 0.1 | 0.99 | 0.002 |
| Mammary tissue | spot | RandomForest | 0.2 | 1.00 | 0.002 |
| Mammary tissue | spot | RandomForest | 0.3 | 1.00 | 0.002 |

**Table S4 continued** — Fur

| Sample type | Training representation | Model | min_det | AUC | Raw p |
|---|---|---|---|---|---|
| Fur | animal-mean | PLS-DA | 0.0 | 0.88 | 0.018 |
| Fur | animal-mean | PLS-DA | 0.1 | 0.91 | 0.006 |
| Fur | animal-mean | PLS-DA | 0.2 | 0.88 | 0.026 |
| Fur | animal-mean | PLS-DA | 0.3 | 0.93 | 0.008 |
| Fur | animal-mean | RandomForest | 0.0 | 0.73 | 0.319 |
| Fur | animal-mean | RandomForest | 0.1 | 0.74 | 0.287 |
| Fur | animal-mean | RandomForest | 0.2 | 0.70 | 0.371 |
| Fur | animal-mean | RandomForest | 0.3 | 0.70 | 0.419 |
| Fur | spot | PLS-DA | 0.0 | 0.76 | 0.184 |
| Fur | spot | PLS-DA | 0.1 | 0.75 | 0.182 |
| Fur | spot | PLS-DA | 0.2 | 0.66 | 0.411 |
| Fur | spot | PLS-DA | 0.3 | 0.55 | 0.796 |
| Fur | spot | RandomForest | 0.0 | 0.74 | 0.305 |
| Fur | spot | RandomForest | 0.1 | 0.73 | 0.281 |
| Fur | spot | RandomForest | 0.2 | 0.74 | 0.249 |
| Fur | spot | RandomForest | 0.3 | 0.69 | 0.365 |

**Table S4 continued** — Liver

| Sample type | Training representation | Model | min_det | AUC | Raw p |
|---|---|---|---|---|---|
| Liver | animal-mean | PLS-DA | 0.0 | 0.58 | 0.792 |
| Liver | animal-mean | PLS-DA | 0.1 | 0.58 | 0.766 |
| Liver | animal-mean | PLS-DA | 0.2 | 0.58 | 0.838 |
| Liver | animal-mean | PLS-DA | 0.3 | 0.58 | 0.741 |
| Liver | animal-mean | RandomForest | 0.0 | 0.38 | 0.872 |
| Liver | animal-mean | RandomForest | 0.1 | 0.38 | 0.906 |
| Liver | animal-mean | RandomForest | 0.2 | 0.38 | 0.856 |
| Liver | animal-mean | RandomForest | 0.3 | 0.38 | 0.878 |
| Liver | spot | PLS-DA | 0.0 | 0.54 | 0.908 |
| Liver | spot | PLS-DA | 0.1 | 0.56 | 0.824 |
| Liver | spot | PLS-DA | 0.2 | 0.54 | 0.816 |
| Liver | spot | PLS-DA | 0.3 | 0.56 | 0.816 |
| Liver | spot | RandomForest | 0.0 | 0.42 | 0.902 |
| Liver | spot | RandomForest | 0.1 | 0.42 | 0.862 |
| Liver | spot | RandomForest | 0.2 | 0.42 | 0.884 |
| Liver | spot | RandomForest | 0.3 | 0.38 | 0.852 |

**Table S4 continued** — Serum

| Sample type | Training representation | Model | min_det | AUC | Raw p |
|---|---|---|---|---|---|
| Serum | animal-mean | PLS-DA | 0.0 | 0.55 | 0.838 |
| Serum | animal-mean | PLS-DA | 0.1 | 0.55 | 0.848 |
| Serum | animal-mean | PLS-DA | 0.2 | 0.55 | 0.850 |
| Serum | animal-mean | PLS-DA | 0.3 | 0.56 | 0.816 |
| Serum | animal-mean | RandomForest | 0.0 | 0.33 | 0.657 |
| Serum | animal-mean | RandomForest | 0.1 | 0.38 | 0.754 |
| Serum | animal-mean | RandomForest | 0.2 | 0.34 | 0.665 |
| Serum | animal-mean | RandomForest | 0.3 | 0.34 | 0.711 |
| Serum | spot | PLS-DA | 0.0 | 0.50 | 1.000 |
| Serum | spot | PLS-DA | 0.1 | 0.53 | 0.984 |
| Serum | spot | PLS-DA | 0.2 | 0.41 | 0.820 |
| Serum | spot | PLS-DA | 0.3 | 0.47 | 0.942 |
| Serum | spot | RandomForest | 0.0 | 0.28 | 0.581 |
| Serum | spot | RandomForest | 0.1 | 0.28 | 0.583 |
| Serum | spot | RandomForest | 0.2 | 0.31 | 0.599 |
| Serum | spot | RandomForest | 0.3 | 0.28 | 0.559 |

**Table S5.** Detection of the ions discussed in the text in mammary tissue: number of tumor-bearing and control animals (of 19) in which each ion was detected, and direction of the animal-level difference (n.s., not significant).

| m/z (feature) | Observed centroid | Mammary tissue | Detected, tumor | Detected, control |
|---|---|---|---|---|
| 52.02 | 52.0271 | lower in tumor | 19/19 | 19/19 |
| 53.02 | 53.0351 | lower in tumor | 19/19 | 19/19 |
| 54.04 | 54.0438 | lower in tumor | 19/19 | 19/19 |
| 65.03 | 65.0356 | lower in tumor | 19/19 | 19/19 |
| 66.02 | 66.0430 | lower in tumor | 19/19 | 19/19 |
| 67.05 | 67.0542 | lower in tumor | 19/19 | 19/19 |
| 78.02 | 78.0422 | lower in tumor | 19/19 | 19/19 |
| 80.06 | 80.0599 | lower in tumor | 19/19 | 19/19 |
| 58.06 | 58.0652 | higher in tumor | 19/19 | 19/19 |
| 104.11 | 104.1077 | higher in tumor | 18/19 | 19/19 |
| 118.07 | 118.0727 | higher in tumor | 17/19 | 2/19 |
| 143.08 | 143.0772 | higher in tumor | 17/19 | 1/19 |
| 145.09 | 145.0942 | higher in tumor | 17/19 | 1/19 |
| 96.92 | 96.9179 | n.s. | 19/19 | 19/19 |
| 112.89 | 112.8905 | higher in tumor | 19/19 | 19/19 |
| 114.90 | 114.8875 | higher in tumor | 19/19 | 19/19 |
| 174.87 | 174.8691 | higher in tumor | 17/19 | 1/19 |

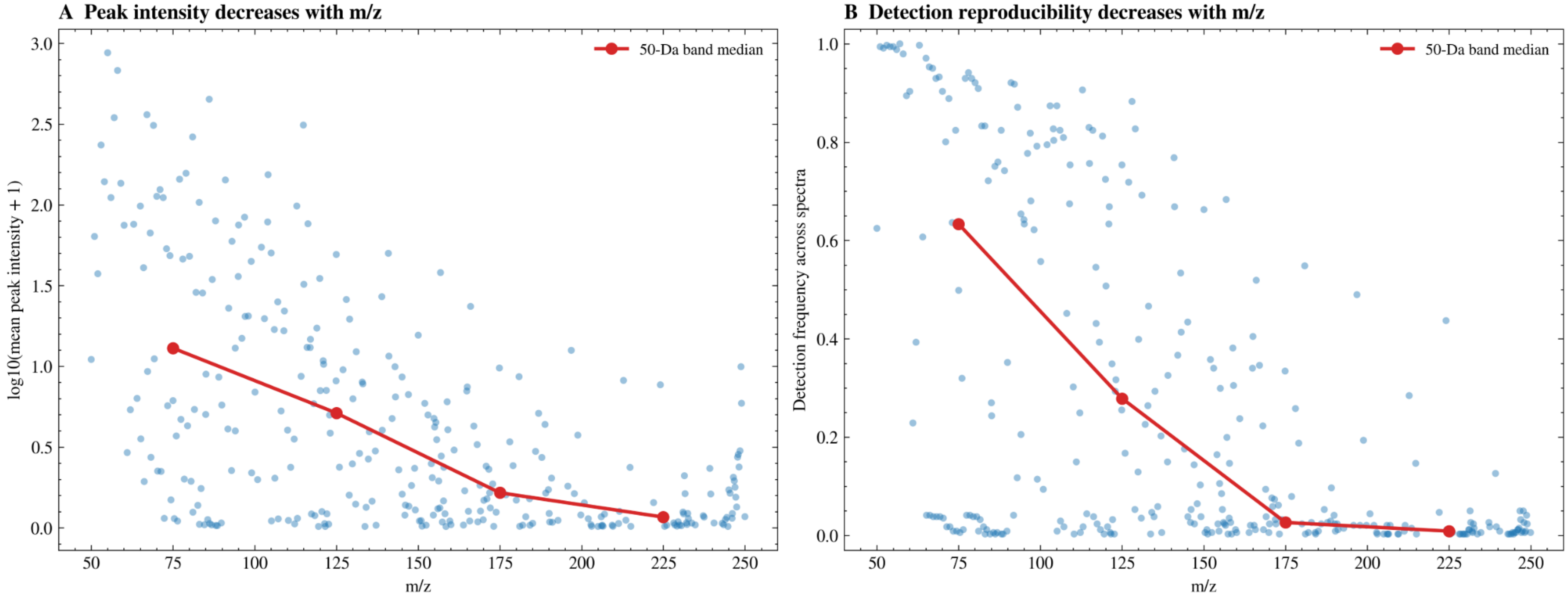


**Figure S1.** Basis for the low-mass (m/z 50–250) analysis window. Across the aligned peak table, (A) mean peak intensity and (B) detection reproducibility (fraction of spectra in which a feature is detected) both decrease markedly with m/z (red line: 50-Da band median). Low-mass ions are therefore more intense and more reproducibly detected than higher-mass ions, supporting the focus on this window.

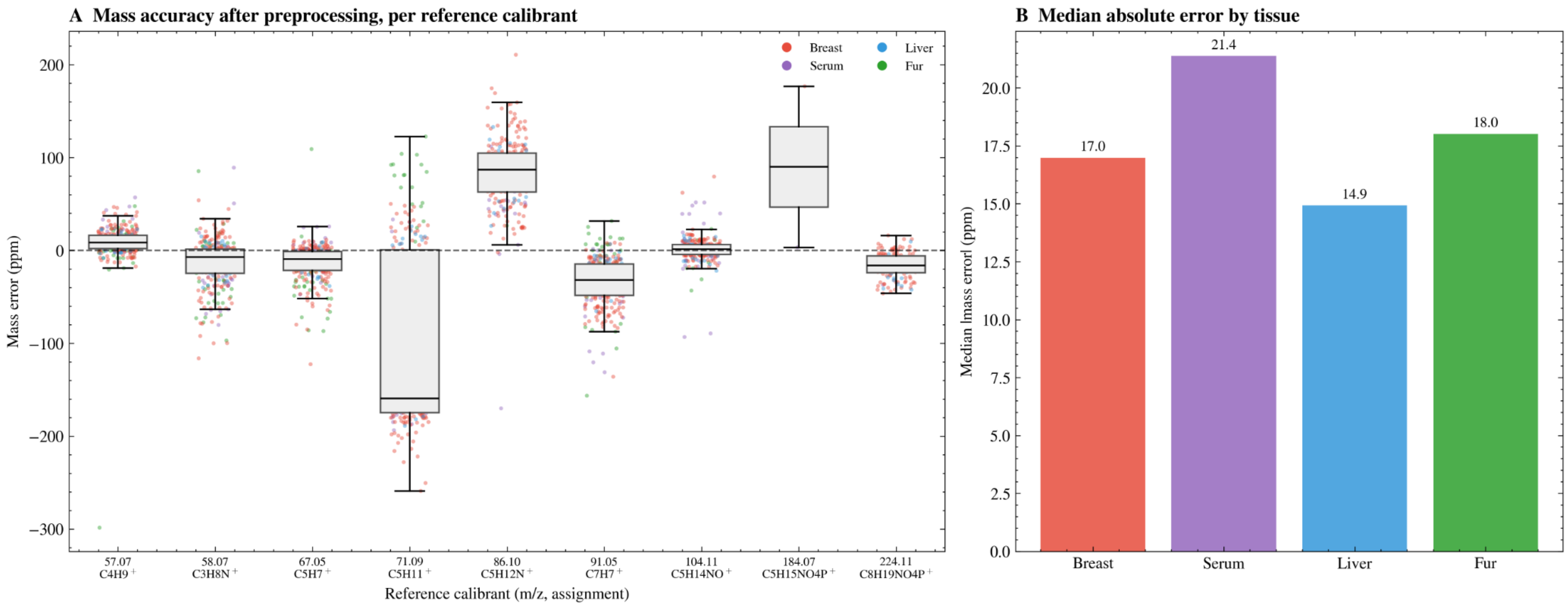


**Figure S2.** Mass-calibration accuracy after preprocessing. For every calibrated/denoised/baseline-corrected/normalised spectrum, the detected peak nearest each reference calibrant in the analysed m/z 50–250 window was matched (within 300 ppm) and its mass error computed. (A) Signed mass error per reference calibrant across all samples (points coloured by tissue). (B) Median absolute mass error per tissue.

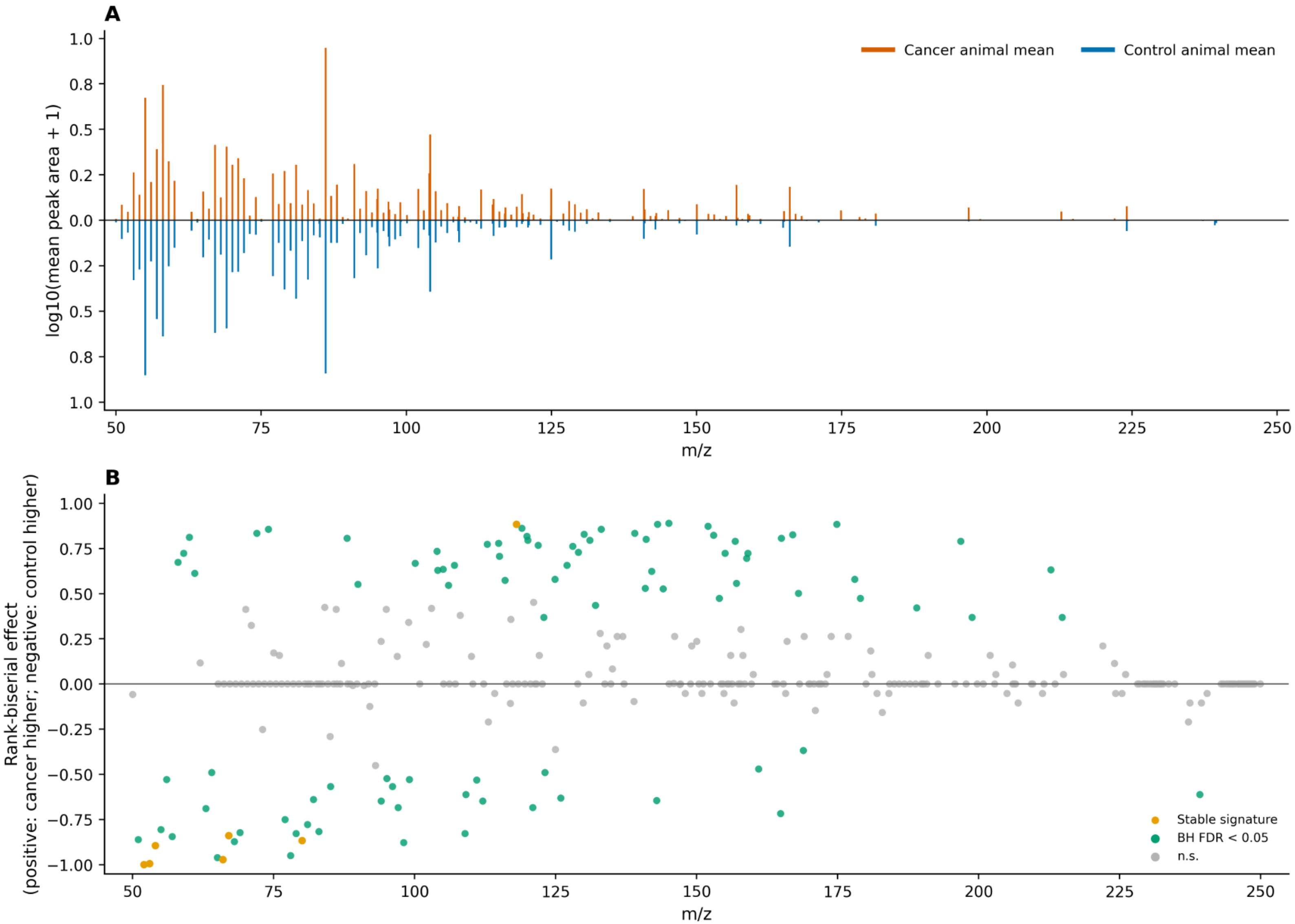

A
Cancer animal mean
Control animal mean
log10(mean peak area + 1)
m/z
B
Rank-biserial effect
(positive: cancer higher; negative: control higher)
Stable signature
BH FDR < 0.05
n.s.
m/z

**Figure S3.** Animal-level ion evidence in mammary tissue. (A) Mirror plot of animal-mean peak-area profiles across the pre-selected *m/z* 50–250 window (cancer upward, control downward). (B) Animal-level rank-biserial effects from two-sided Mann–Whitney tests; green points meet BH FDR<0.05 across 313 ions and orange points denote the stable signature (ions selected in ≥90% of cross-validation folds by both random forest and univariate testing).

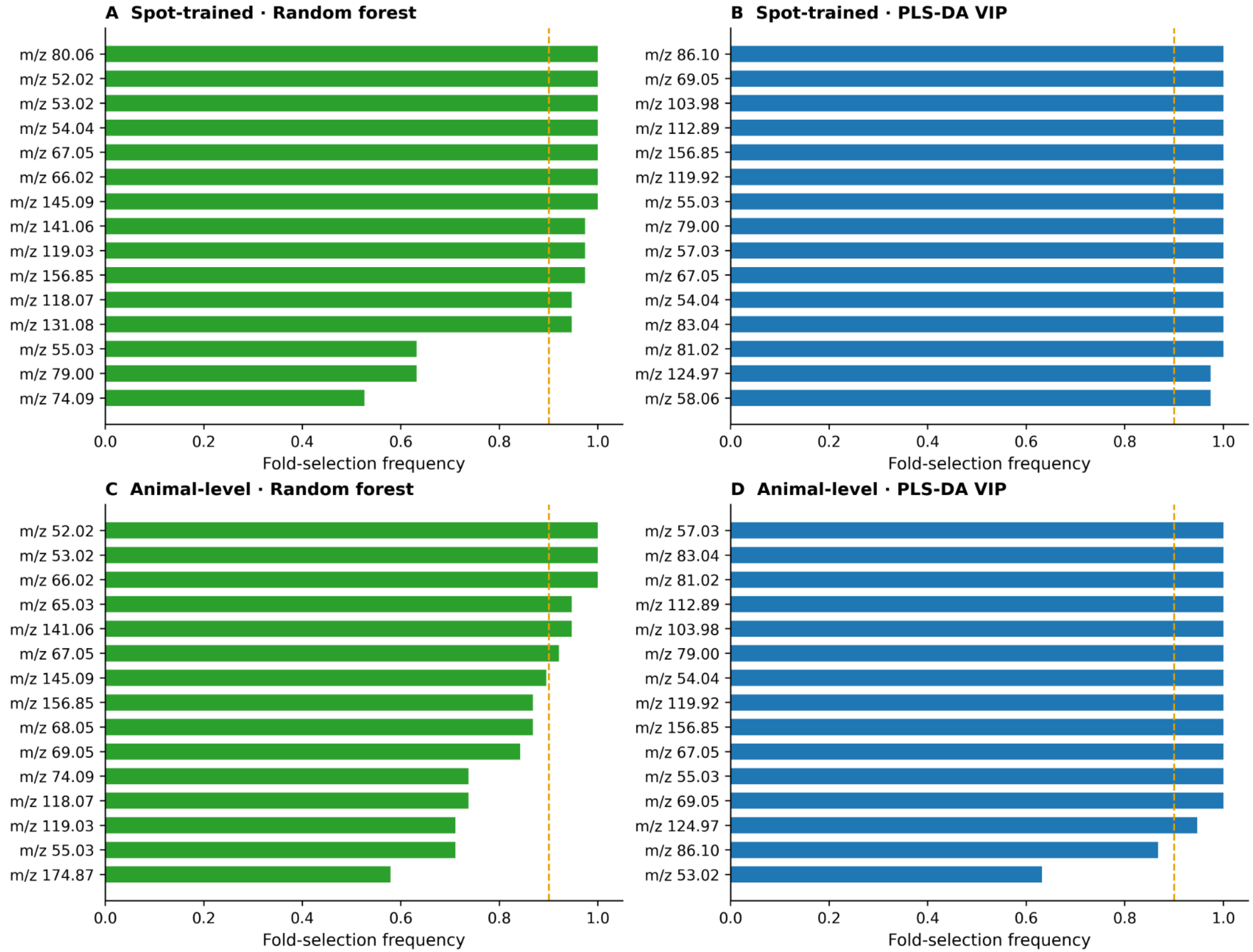

**Figure S4.** Cross-validation stability of the mammary ion signature. In each LOAO training fold the top 15 ions were recorded by each criterion; panels show the fold-selection frequency of the top 15 ions, sorted in descending order, for (A) spot-trained random forest, (B) spot-trained PLS-DA VIP, (C) animal-level random forest, and (D) animal-level PLS-DA VIP. The dashed line marks 90%.

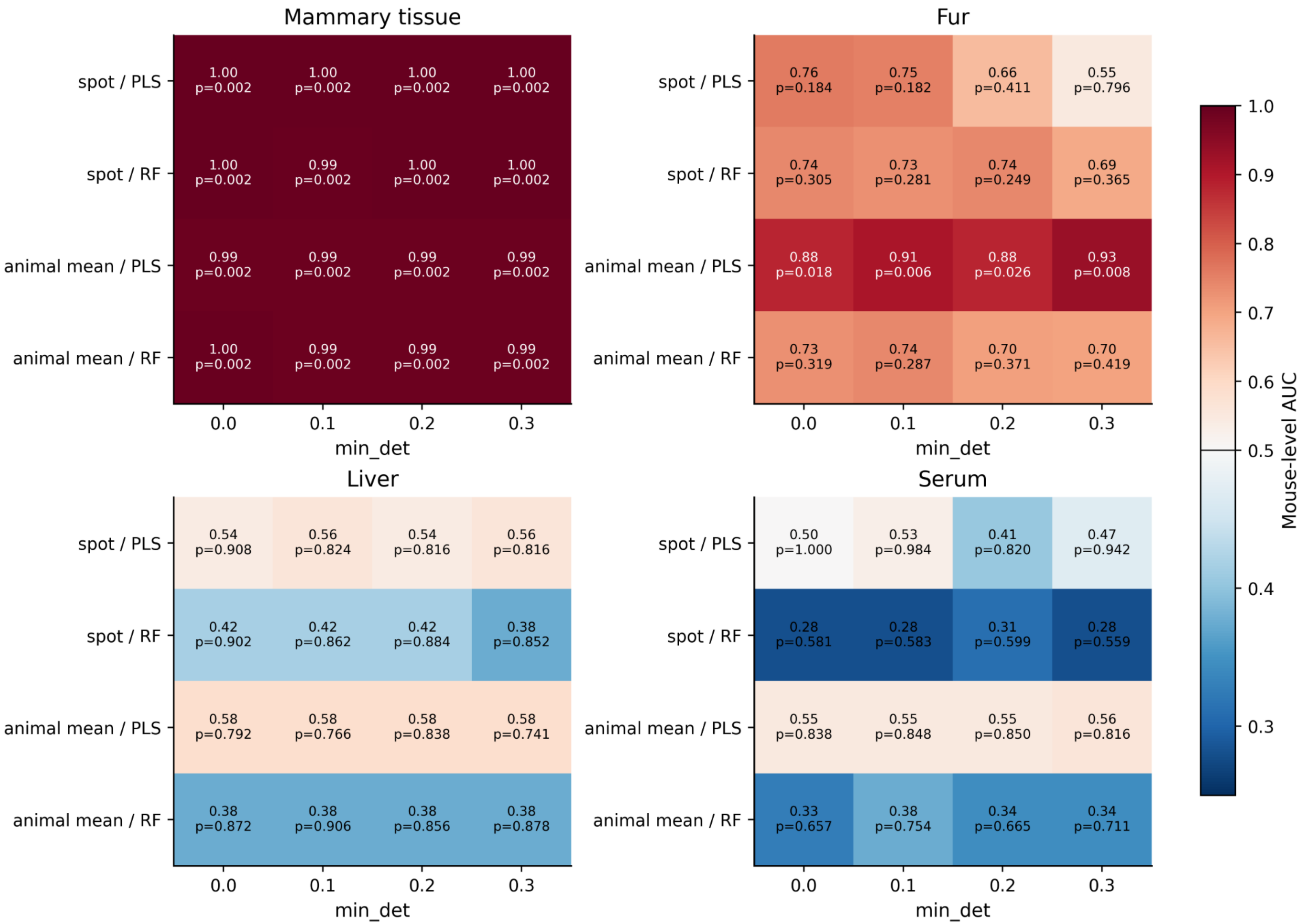


**Figure S5.** Exploratory sensitivity grid. Cells show mouse-level AUC and animal-label permutation p values (mammary p≈0.002, floored by its near-perfect AUC; peripheral p from 500 permutations). The grid assesses robustness to analytical choices and does not create new confirmatory claims.

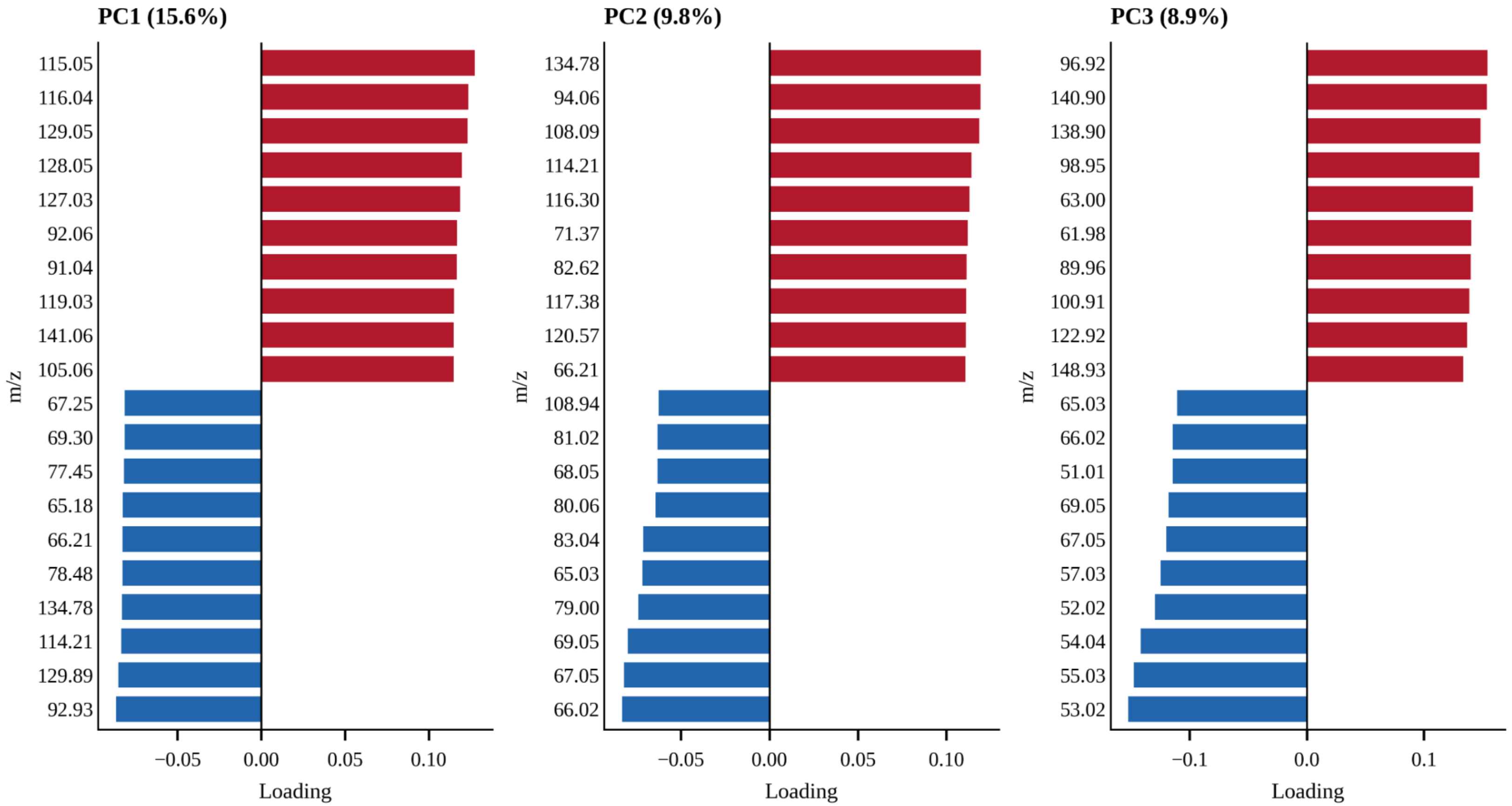


**Figure S6.** Loadings of the PCA shown in Figure 3 (ten most positive and ten most negative per component).

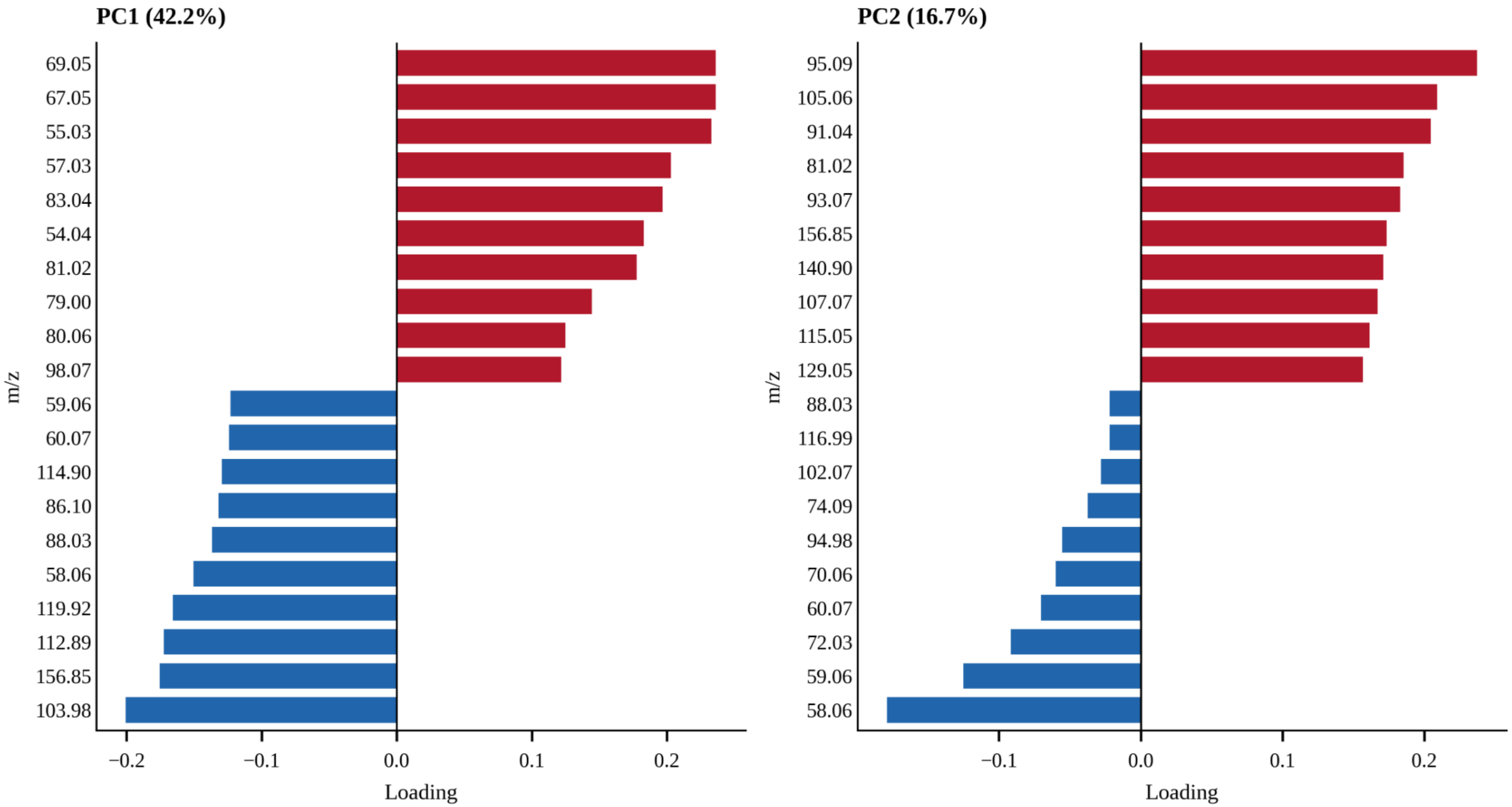


**Figure S7.** Loadings of the mammary-tissue PCA shown in Figure 2C (ten most positive and ten most negative per component).
